# Splenic artery pseudoaneurysm presenting as massive haematemesis during endoscopic management of post-sleeve gastrectomy leak: a case report

**DOI:** 10.1093/jscr/rjag782

**Published:** 2026-09-04

**Authors:** Safiya Al-Masrouri, Abdullah Al Jabri, Khalid Al Shamousi, Ruqaiya Al Shehhi, Humaid AlAdawi

**Affiliations:** Department of Surgery, College of Medicine and Health Sciences, Sultan Qaboos University, PO Box 35, Al Khoud, Muscat 123, Oman; Department of Surgery, Sultan Qaboos University Hospital, PO Box 35, Al Khoud, Muscat 123, Oman; Department of Medicine, College of Medicine and Health Sciences, Sultan Qaboos University, PO Box 35, Al Khoud, Muscat 123, Oman; Department of Surgery, Sultan Qaboos University Hospital, PO Box 35, Al Khoud, Muscat 123, Oman; Department of Surgery, Sultan Qaboos University Hospital, PO Box 35, Al Khoud, Muscat 123, Oman

**Keywords:** sleeve gastrectomy, splenic artery pseudoaneurysm, gastrointestinal haemorrhage, coil embolization, diagnostic anchoring

## Abstract

Visceral artery pseudoaneurysm is a rare, potentially fatal complication of post-sleeve gastrectomy (LSG) staple-line leak. We report a 36-year-old woman who presented with a proximal staple-line leak, managed with laparoscopic washout and endoscopic stenting. During apparent clinical improvement, she experienced sentinel haemorrhage on postoperative Day 30, initially attributed to stent-related mucosal trauma. Catastrophic haemorrhage ensued 2.5 hours later. Endoscopy revealed a clot-filled stent without a mucosal source; computed tomography angiography (CTA) identified a 20 × 18 × 13 mm splenic artery pseudoaneurysm within the perigastric collection. Proximal coil angioembolization achieved complete exclusion without splenic infarction. The patient made a full recovery and was discharged home on postoperative Day 42. This case illustrates a potentially fatal vascular complication arising during active leak management. A splenic artery pseudoaneurysm must be actively sought in any upper gastrointestinal bleeding during post-LSG leak management, and CTA must exclude a vascular source before attributing bleeding to stent-related mucosal trauma.

## Introduction

Staple-line leak complicates 2.4% of laparoscopic sleeve gastrectomy (LSG) procedures, with 89% arising at the proximal sleeve [1]. Management involves a staged, multidisciplinary approach encompassing source control, nutritional optimization, and endoscopic therapies [2, 3]. Endoscopic strategies include covered self-expanding metal stents (SEMS) and endoscopic internal drainage (EID) via transgastric double-pigtail stents, achieving leak closure in up to 83% of cases [3]. As endoscopic management has expanded, prolonged perigastric inflammation creates conditions for rare vascular complications. Visceral artery pseudoaneurysm (VAP) is a rare vascular complication in this setting, often presenting with a deceptively minor sentinel bleed before catastrophic rupture [4]. In the largest dedicated series, VAP accounted for 75% of upper gastrointestinal bleeding complicating post-LSG fistula, and primary angiography was recommended over endoscopy when VAP is suspected in haemodynamically stable patients [5]. We report a case of splenic artery pseudoaneurysm (SAP) arising during active multimodal leak management, illustrating a critical and preventable diagnostic pitfall.

## Case report

A 36-year-old woman (body mass index 38 kg/m^2^) with a history of recurrent pre-eclampsia, an obesity-associated risk factor with no other recognized relevance to this presentation, underwent LSG at an outside institution. On postoperative Day (POD) 13 (all POD references are from the index LSG), she presented with sepsis: fever (38.5°C), tachycardia (110 beats/min), leukocytosis of 16 × 10^9^/L, CRP 220 mg/L, and haemoglobin 110 g/L. Computed tomography (CT) demonstrated a 7 × 6 × 5 cm gas-containing perigastric collection medial to the spleen, consistent with a proximal staple-line leak; percutaneous drainage was precluded by colonic interposition.

She was admitted overnight for resuscitation with intravenous antibiotics and fluids, then underwent urgent laparoscopic washout the following morning (POD 14), evacuating 100 mL of pus. A 1.5 cm staple-line defect was identified; primary repair was deferred given tissue friability. Two Jackson-Pratt (JP) drains were placed. The procedures were staged because a covered SEMS of appropriate size was not immediately available at our centre and required interval procurement. On POD 15, upper endoscopy confirmed the defect and a covered SEMS (Taewoong, 12 cm) was deployed across the gastroesophageal junction. EID was established via two transgastric double-pigtail stents (7 Fr × 7 cm). Total parenteral nutrition was commenced. Over POD 15–28, fever, tachycardia, and inflammatory markers resolved.

In the early morning of POD 30, the patient experienced a small-volume sentinel haematemesis. Given stable haemodynamics and an indwelling SEMS, the episode was attributed to stent-related mucosal trauma. CT angiography (CTA) was not performed, and an upper endoscopy was planned.

**Figure 1:**
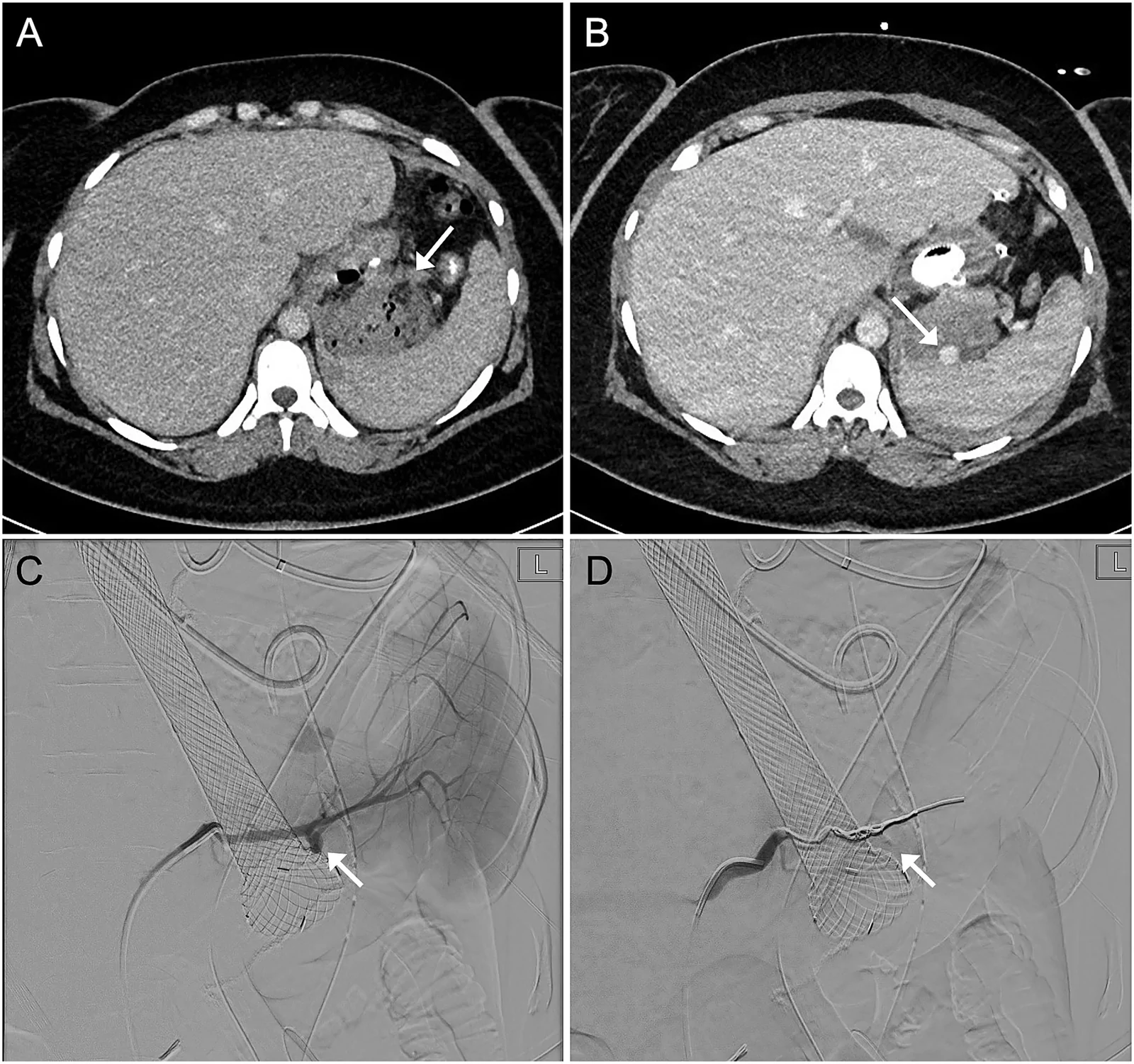
Imaging and endovascular management of splenic artery pseudoaneurysm. (A) CT (POD 13): 7 × 6 × 5 cm gas-containing perigastric collection medial to spleen (arrow). (B) CTA (POD 30): 20 × 18 × 13 mm saccular SAP, distal splenic artery (arrow); no extravasation; splenic perfusion preserved. (C) Pre-embolization arteriography: SAP confirmed (arrow); no active blush. (D) Post-embolization: complete pseudoaneurysm exclusion after proximal coil embolization (arrow).

Two and a half hours later, the patient developed massive haematemesis with haemodynamic compromise (tachycardia 130 beats/min; BP 115/60 mmHg). Haemoglobin fell to 70 g/L; resuscitation required 5 units packed red blood cells, 5 units fresh frozen plasma, and 5 units platelets. Endoscopy demonstrated a clot-filled SEMS without mucosal source. Immediate CTA identified a 20 × 18 × 13 mm saccular pseudoaneurysm of the distal splenic artery within the perigastric collection; splenic perfusion was preserved with no active extravasation. The patient was transferred directly to the angiography suite, where multiple coils were deployed at the proximal splenic artery via right femoral access, achieving complete angiographic exclusion by inflow occlusion while preserving distal perfusion via short gastric collaterals (Fig. 1). The patient was admitted to the intensive care unit overnight without further haemorrhage.

On POD 31 (i.e. one day after embolisation), laparoscopic re-exploration evacuated 500 mL of clotted blood; the spleen was viable. The patient improved steadily with no further bleeding. By POD 42, CT confirmed abscess resolution, pseudoaneurysm exclusion, and no splenic infarction. The SEMS and EID stents were removed; fluoroscopy confirmed complete leak closure without stenosis. At one year, the patient achieved 70% excess weight loss with normal nutritional parameters. She subsequently developed post-traumatic stress disorder requiring psychiatric follow-up.

## Discussion

VAP complicating a post-LSG leak is rare, with fewer than 20 cases reported in the literature, yet carries life-threatening potential [5, 6]. Published cases span days to years after surgery, summarized in Table 1 [5, 12, 13]. In all but the present case, vascular complications presented remote from the index leak episode; the present case is distinctive in that SAP arose during active concurrent surgical and endoscopic management, a sequence not previously described. The predominant mechanism is enzymatic erosion of the splenic artery wall by an adjacent infected perigastric collection. In this case, the index CT demonstrated the collection lying directly medial to the spleen, placing the distal splenic artery within the zone of ongoing inflammatory injury throughout management—a finding that, in retrospect, warranted vascular vigilance from the outset. Despite this, the central failure was not the pseudoaneurysm itself but its misattribution. A sentinel bleed—a minor, self-limiting haemorrhage preceding catastrophic arterial rupture—is a well-recognized vascular phenomenon, but its significance is easily underestimated when a familiar alternative explanation is at hand [4]. In this case, haematemesis with new-onset epigastric pain was attributed to stent mucosal trauma and CTA deferred—a textbook example of diagnostic anchoring. Three features should have raised suspicion: a perigastric collection proximate to the splenic artery on index CT, new-onset pain, and absence of prior mucosal symptoms despite weeks of indwelling SEMS. Stent mucosal injury is a genuine cause of bleeding [5], but must remain a diagnosis of exclusion after vascular imaging. From a diagnostic standpoint, any upper gastrointestinal bleeding in a haemodynamically stable patient during post-LSG leak management should prompt immediate CTA rather than endoscopy alone. CTA provides high diagnostic sensitivity and the anatomic roadmap essential for procedural planning [4]. The absence of active extravasation should not be reassuring—intermittent bleeding with spontaneous tamponade is well recognized [4]—and a clot-filled stent without a mucosal source is characteristic of extraluminal arterial haemorrhage. In retrospect, proceeding with endoscopy may have delayed definitive vascular management. Fortunately, the patient remained haemodynamically stable throughout, allowing seamless transfer from the endoscopy suite directly to CT angiography and then to interventional radiology. Vascular injury is not limited to the splenic artery; left gastric pseudoaneurysm has also been reported [5, 9], underscoring the need for a broad arterial differential when endoscopy is non-diagnostic. Once identified, transcatheter embolization is the first-line intervention in stable patients, consistent with Society for Vascular Surgery guidelines [14, 15]. Angioembolization has been successful across a range of presentations [5, 12, 13], though surgical intervention remains essential for instability or endovascular failure. Reported mortality in this setting has followed both failed embolization and delayed surgical intervention [5, 6], reinforcing that early vascular diagnosis is the determinant of survival. This patient also developed post-traumatic stress disorder requiring psychiatric follow-up. Patients surviving near-fatal bariatric complications carry a substantial psychological burden that is often underrecognized amid the focus on physical recovery; routine psychological screening should be incorporated into structured follow-up for patients experiencing life-threatening complications in this setting.

**Table 1:** Summary of reported post-sleeve gastrectomy visceral artery pseudoaneurysm and related vascular complications.

Author (Year)	n	Time from LSG	Leak/fistula	Presentation	Management	Outcome
Rebibo *et al*. (2013) [5]	4	Median 15 d from fistula dx^a^	Gastric fistula (all)	Massive UGIB	Coil embolization (*n* = 1); failed embolization → laparotomy (*n* = 1); laparotomy + splenectomy (*n* = 1); stent removal + laparotomy (*n* = 1)	3 survival; 1 mortality^b^
Ball *et al*. (2018) [7]	1	6 months	Gastrocolonic fistula	UGIB	Coil embolization	Survival
Montana *et al*. (2018) [6]	3	16 d–5 yr	Gastrosplenic fistula (all)	Massive UGIB	Embolization (*n* = 1); laparotomy + splenectomy (*n* = 2)	2 survival; 1 mortality
Ricci *et al*. (2019) [8]	1	2 years	Suspected chronic fistula	Massive UGIB	Laparotomy + splenectomy	Survival
Berjawi *et al*. (2020) [9]	1	3 weeks	No	Massive UGIB	Coil embolization (left gastric artery)	Survival
Copin *et al*. (2021) [10]	1	5 years	No	Massive UGIB	Coil embolization	Survival
Peters *et al*. (2021) [11]	2	2–4 years	No	Abdominal pain + shock (*n* = 1); pleuritic pain + shock (*n* = 1)	Laparotomy + splenectomy (*n* = 1); coil embolization (*n* = 1)	All survival
Najjari *et al*. (2021) [12]	1	3 years	No	Pain + haemorrhagic shock	Laparotomy + splenectomy	Survival
Babic & Ramachandran (2024) [13]	1	2 years	No	Pain + haemorrhagic shock	Laparotomy + splenectomy	Survival

^a^Rebibo *et al*. reported time from fistula diagnosis, not from index LSG.

^b^One patient (left gastric artery pseudoaneurysm, failed embolization) suffered cardiac arrest and died from anoxic brain injury. LSG, laparoscopic sleeve gastrectomy; POD, postoperative day; UGIB, upper gastrointestinal bleeding.

## Conclusion

SAP can arise during post-LSG leak management. In haemodynamically stable patients, any upper gastrointestinal bleeding in this setting demands CTA to exclude a vascular source before attributing haemorrhage to stent-related mucosal trauma. Angioembolization is the treatment of choice in stable patients.
